# Prognostic Factors Predicting Poor Outcome in Cancer Patients with Febrile Neutropenia in the Emergency Department: Usefulness of qSOFA

**DOI:** 10.1155/2018/2183179

**Published:** 2018-10-11

**Authors:** Seung Jae Lee, Ji Hye Kim, Seung Baik Han, Jin Hui Paik, Areum Durey

**Affiliations:** Department of Emergency Medicine, Inha University School of Medicine, Incheon, Republic of Korea

## Abstract

**Background/Aims:**

Febrile neutropenia is considered as one of the most important and potentially life-threatening oncologic emergencies, which requires prompt medical assessment and treatment with antibiotics. This was a single-center retrospective study that investigated the prognostic factors predicting poor outcome in patients with cancer who presented with febrile neutropenia at the emergency department (ED).

**Methods:**

The medical records of patients diagnosed with febrile neutropenia in the ED from January 2014 to December 2017 were reviewed. Patients aged >18 years who were diagnosed with a malignancy were included in the analysis. Febrile neutropenia was defined as an absolute neutrophil count < 1,000/mm^3^ with a temperature greater than 38°C. Patients were divided into two groups: those who were admitted at the intensive care unit (ICU) or those who died in the hospital (case group) and those who were admitted at general wards and were discharged (control group). The two groups were compared to determine the factors associated with poor prognosis.

**Results:**

We identified 104 patients (25 and 79 from the case and control groups, respectively) with cancer who presented with febrile neutropenia at the ED during the study period. Lower blood pressure, platelet count, and HCO_3_^−^ level, higher CRP and creatinine level, and the presence of bacteremia were more commonly observed in the case group than in the control group. In the multiple logistic regression analysis, the following independent predictors significantly correlated with ICU admission and in-hospital mortality: quick sequential (sepsis-related) organ failure assessment (qSOFA) score (odds ratio [OR]: 4.62; 95% confidence interval [CI]: 1.17–18.22; p=0.285), hemoglobin level (OR: 0.51; 95% CI: 0.33–0.78; p=0.002), total bilirubin level (OR: 7.69; 95% CI: 1.29–45.8; p=0.025), and respiratory tract infection (OR: 29.65; 95% CI: 3.81–230.7; p=0.0012).

**Conclusions:**

The qSOFA can be a useful bedside tool for patients with cancer who present with febrile neutropenia at the ED. Moreover, it can help emergency physicians in identifying patients at risk of poor prognosis and in initiating prompt empirical antimicrobial therapy. Further studies must be conducted to validate the efficacy of the qSOFA in these patients in the ED.

## 1. Introduction

Neutrophils constitute the main mechanism of host defense against infection and serve as an essential component of innate immunity. The degree and duration of neutropenia were identified as key factors related to the risk and outcome of infection in 1979 [1] and it is as true today as when it was written [2, 3]. Therefore, febrile neutropenia is considered as one of the most important and potentially life-threatening oncologic emergencies, which requires prompt medical assessment and treatment with antibiotics [4].

However, patients with febrile neutropenia may be a heterogeneous group in terms of medical complications and mortality, with a limited number of patients developing serious medical complications [5], and the identification of patients at risk is challenging for physicians. Notably, a prospective study in France has revealed that the severity was under-evaluated, and the management of patients with cancer who present with febrile neutropenia at the ED is insufficient [6].

This study aimed to identify the prognostic factors predicting poor outcome in patients with cancer who presented with febrile neutropenia at the ED. Moreover, the characteristics and outcomes in these patients were retrospectively analyzed, and the independent variables that can be easily assessed and used at the ED to predict patients who are at risk of developing potentially serious complications were identified.

## 2. Material and Methods

### 2.1. Study Design

This was a single-center retrospective study that investigated the prognostic factors predicting poor outcome in patients with cancer who presented with febrile neutropenia at the ED from January 2014 to December 2017. This study was conducted in a university hospital in Korea, which is a tertiary hospital with 60,000 patients according to an annual census of ED visits, and was approved by the institutional review board of the hospital (IRB no. 2018-05-004). The need for informed consent was waived.

### 2.2. Study Protocol and Population

The medical records of patients diagnosed with febrile neutropenia at the ED during the 5-year study period were reviewed from the computer database. Patients with malignancies and those who were older than 18 years were included. Febrile neutropenia was defined as an absolute neutrophil count (ANC) < 1,000/mm^3^ with a temperature greater than 38°C. Only the first episode of febrile neutropenia in a patient during the study period was considered.

Patients were divided into two groups: those who were admitted at the intensive care unit (ICU) or those who died during hospitalization (case group) and those who were admitted at general wards and were discharged (control group). The two groups were compared to determine the factors associated with poor prognosis.

### 2.3. Variables

The following information on the case and control groups was obtained by reviewing the medical charts: age, sex, comorbidities, clinical manifestations (ED visit on weekends, change in mental status, duration of fever [> 24 h], presence of central venous catheter, and hospital-acquired infection), type and origin of malignancy, and history of chemotherapy. Vital signs at triage and laboratory results, including microbiologic test results, were recorded, and the quick sequential (sepsis-related) organ failure assessment (qSOFA) score was calculated. The presumed source of infection, empirical antimicrobial therapy, time from ED visit to the administration of antibiotics, appropriateness of the empirical therapy, and use of granulocyte colony-stimulating factor were identified. Clinical outcome variables, such as vasopressor use, need for mechanical ventilation, ICU admission, do not resuscitate (DNR) order, and duration of hospitalization, were also documented.

### 2.4. Definitions

The time from fever onset to ED admission was defined as the time from the onset of subjective symptoms (based on patient history) to ED visit. Central venous catheter included medication ports and peripherally inserted central catheters. The qSOFA score included a systolic blood pressure (BP) ≤ 100 mmHg, RR ≥ 22/minute, and altered mental status [7]. Each of the above-mentioned conditions corresponded to one point, and the score ranged from 0 to 3. Profound neutropenia is defined as an ANC lower than 100/mm^3^ upon ED arrival.

When focal infection could not be identified, the source was categorized as undetermined. The time from ED visit to the administration of antibiotics is defined as the time in minutes from presentation to triage to the first dose of parental antibiotics and is considered as a continuous variable. The initial empirical antimicrobial therapy was considered appropriate if the initial antibiotics included at least one antibiotic that was active in vitro and if the dosage was in accordance with current medical standards. Otherwise, initial antimicrobial therapy was considered inappropriate.

### 2.5. Statistical Analyses

Data with a normal distribution were expressed as mean ± standard deviation and were analyzed using the independent samples t-test. Data with a skewed distribution were expressed as medians and interquartile ratios and were analyzed using the Mann–Whitney U test. Categorical variables were compared using *χ*^2^ test or Fisher exact test depending on the sample size. A simple logistic regression analysis followed by a stepwise multiple logistic regression analysis was performed to identify discriminative variables between the groups at the ED. A* p* value < 0.05 was considered statistically significant. All statistical analyses were performed using MedCalc for Windows version 17.6 (MedCalc Statistical Software, Ostend, Belgium).

## 3. Results

### 3.1. Clinical Characteristics

We identified 104 patients with cancer who presented with febrile neutropenia at the ED during the study period. The case group consisted of 25 patients who were admitted at the ICU or those who died during hospitalization, whereas the control group included the remaining 79 patients who were admitted at general wards and were discharged. Demographic and clinical data comparing the groups are presented in Table 1.

The mean age of the participants was 61 years, and diabetes mellitus was the most frequent comorbidity (18%). Approximately 35% of the patients visited the ED during weekends, and 32% had fever for more than 24 h prior to their visit. Moreover, 16% of the patients had central venous line in place, and 9% presented with hospital-acquired infection. No statistical difference was observed between the groups in terms of comorbidities, ED visit during weekends, duration of fever, presence of a central venous line, and hospital-acquired infection.

Of the 104 patients, 69 had a solid tumor, and 35 presented with hematological malignancies. Among the solid tumors, the most frequent origin was the breast, followed by the gastrointestinal tract and the lungs. Around 49% (34 out of 69) of the patients presented with stage IV solid tumor. Leukemia occurred in 11% of the patients.

Approximately 89 patients (18 and 71 from the case and control groups, respectively) received chemotherapy within 2 months, and the median delay between chemotherapy and ED visit was 12.5 days, ranging from 10 to 15 days.

### 3.2. Vital Signs and Laboratory Findings

Table 2 shows the vital signs and laboratory findings of the patients upon ED visit. Patients who died or those who were admitted at the ICU had lower systolic and diastolic BP and higher pulse rates than those in the control group. Notably, the mean value of the qSOFA was significantly higher in the case group than in the control group (0.88 vs. 0.36, p=0.0003). With regard to complete blood cell counts, no difference was observed between the groups in terms of ANC or profound neutropenia. However, the case group had a lower hemoglobin level, platelet count, and HCO_3_^−^ level and a higher total bilirubin, creatinine, and C-reactive protein (CRP) level than the control group.

### 3.3. Source of Infection and Microbiology

The presumed source of infection and the results of the microbiologic study are summarized in Table 3. With regard to the source of infection, pneumonia was more common in patients who died or those who were admitted at the ICU (52% vs. 16%, respectively; p=0.0004), whereas undetermined origin was more frequently observed in the control group than in the case group (66% vs. 24%, respectively; p=0.0003)

Bacteremia was more frequently observed in the case group than in the control group (24% vs. 8%, respectively; p=0.035). Among the case patients, 6 had bacteremia; 2 presented with* Escherichia coli*, 2 with* Pseudomonas aeruginosa*, 1 with* Klebsiella pneumoniae*, and 1 with* Staphylococcus aureus*. No resistant bacteria, such as extended-spectrum *β*-lactamase (ESBL)-producing* Enterobacteriaceae*, multidrug-resistant* P. aeruginosa,* or methicillin-resistant* S. aureus,* were observed in the case group. Among the control patients, 6 had bacteremia; 3 presented with* E. coli*, 1 with* K. pneumoniae*, 1 with* Enterobacter*, and 1 with* Streptococcus agalactiae*, of which 2 tested positive for ESBL.

### 3.4. Treatment and Outcome

Table 3 shows the treatment at the ED and clinical outcomes. With regard to antimicrobial treatment, 20% of the patients were treated with combination therapy. Anti-pseudomonal *β*-lactam antibiotics (cefepime and piperacillin tazobactam) were used empirically in 65% and 87% of the case and control patients, respectively. The median time from ED presentation to the start of antibiotic therapy was 107 minutes, and no difference was observed between the groups in terms of the latency of the first dose of antibiotics.

With regard to clinical outcomes, vasopressor was used in 15% of the patients and in 6% of the patients who were intubated at the ED. Moreover, 20 out of the 104 (20%) patients were admitted at the ICU. The in-hospital mortality rate was 12% (12 out of 104 patients), and 5 patients with DNR order were not admitted at the ICU and then died within 24 hours of ED visit.

### 3.5. Predictive Factors for ICU Admission or Mortality

Table 4 shows the variables associated with poor prognosis in patients with cancer who presented with febrile neutropenia at the ED based on a simple logistic regression analysis. In the multiple logistic regression analysis, the independent predictors significantly correlated with ICU admission, and mortality was significantly associated with the qSOFA (odds ratio [OR]: 4.62; 95% confidence interval [CI]: 1.17–18.22; p=0.285), hemoglobin level (OR: 0.51; 95% CI: 0.33–0.78; p=0.002), total bilirubin level (OR: 7.69; 95% CI: 1.29–45.8; p=0.025), and respiratory tract infection (OR: 29.65; 95% CI: 3.81–230.7; p=0.0012)

## 4. Discussion

Two models were used to predict the outcome of febrile neutropenic episode. Talcott et al. [8] have established a clinical prediction rule based on clinical features; however, the model requires information that included tumor response to chemotherapy, which is not easily determined at the ED. Then, the Multinational Association of Supportive Care in Cancer (MASCC) developed a scoring system based on seven independent prognostic factors [5]. The MASCC risk-index score has been internationally validated under various clinical conditions [9, 10]. However, it includes variables, such as burden of illness, severity of symptoms, and history of fungal infection, which could be obtained only through a detailed review of the patient's medical history or might be affected by the subjective judgement of the physician. Therefore, the use of the models is limited in the ED.

To the best of our knowledge, only a few studies [11–13] that aimed to identify independent factors associated with the serious complications of febrile neutropenia in patients with cancer have been conducted in ED settings. In 2009, Moon et al. have reported that laboratory parameters, such as a platelet count < 50,000/mm^3^, serum CRP level > 10 mg/dL, and pulmonary infiltration on chest radiography, were independent factors that can predict the development of complications [12]. In 2013, Lynn et al. have also shown the association of pneumonia, a platelet count ≤ 50,000/mm^3^ and the latency of the first dose of antibiotics in the ED with serious complications [13]. Our study also aimed to identify the risk factors that can predict ICU admission and mortality in patients with cancer who presented with febrile neutropenia at the ED. Nevertheless, there are two important differences from the others. Unlike other studies that only enrolled patients with an ANC < 500/mm^3^, the present study included patients with an ANC between 500/mm^3^ and 1,000/mm^3^. The risk of developing clinically important infection increases with the decrease in neutrophil count below 500/mm^3^. Moreover, individuals with prolonged neutropenia (> 7 days) are at higher risk [14]. However, this is not significant for emergency physicians because neither the duration of nor the course of neutropenia is predictable in patients with cancer who visit the ED, and this can only be determined retrospectively. Therefore, we included patients with neutropenia (ANC < 1,000/mm^3^) [15] to prevent overlooking patients who are at risk. Surprisingly, 4 of the 12 patients who died in our study had an ANC > 500/mm^3^.

The second difference is that our study did not only focus on chemotherapy-induced neutropenia. In fact, it included all causes of neutropenia in patients with cancer. Neutropenia can develop in patients with cancer because of the disease itself that involves the bone marrow. However, severe infection can also cause neutropenia. Because the present study aimed to identify the prognostic factors of febrile neutropenia, which were generally applicable to patients with cancer who visit the ED, we included all causes of neutropenic episodes. In fact, 15 out of 104 patients in our study presented with neutropenia despite the absence of recent chemotherapy within 2 months, of which 8 patients were either admitted at the ICU or died. Consequently, the possibility of febrile neutropenia and the subsequent development of complications should still be considered in patients with cancer even without the history of recent chemotherapy.

Based on our study, the independent predictors significantly correlated with ICU admission, and in-hospital mortality was associated with the qSOFA score, hemoglobin level, total bilirubin, and respiratory tract infection. The qSOFA is a new screening tool for sepsis that has a prognostic performance equal to the full SOFA for patients with suspected infection outside the ICU [7]. According to our study, patients with cancer who presented with febrile neutropenia and who are likely to have a poor prognosis can be rapidly identified at the bedside with the qSOFA. Interestingly, other variables associated with unfavorable outcomes in our study were also the components of the full SOFA, which include hypotension, platelet count, and bilirubin and creatinine level.

This study has several limitations. First, this is single-center retrospective study. Moreover, it is possible that the significant prognostic factors related to ICU admission or in-hospital mortality were not identified due to the small sample size of the study. For instance, other studies have identified chronic obstructive pulmonary disease (COPD) [16], leukemia [17], polymicrobial bacteremia [18], and inappropriateness of antimicrobial therapy [19] as independent risk factors. However, only 8%, 11%, and 12% of the enrolled patients in our study had COPD, leukemia, and bacteremia, respectively. The appropriateness of using empirical antibiotics was only evaluated in 17% of the study population.

Emergency physicians must identify patients who are at risk because the prognosis of patients with severe infection depends on their initial management. The qSOFA and the presence of pneumonia can be a useful bedside tool for patients with cancer who present with febrile neutropenia in the ED. The results of the present study may help emergency physicians identify high-risk patients, thus preventing the development of complications by ordering prompt blood culture and subsequently administering broad-spectrum antibiotics.

## Figures and Tables

**Table 1:** Comparison of clinical characteristics of 104 cancer patients presented with febrile neutropenia to the emergency department including 25 case patients who admitted to the intensive care unit or died in the hospital and 79 control patients who admitted to general wards and discharged.

Characteristics	Total (n = 104)	Case (n = 25)	Control (n = 79)	P value
Age	60.8 ± 13.6	63.6 ± 12.9	61 (51-69)	0.255
Male, no. (%)	41 (39)	14 (56)	27 (34)	0.052

Comorbid conditions, no. (%)				
Diabetes mellitus	14 (13)	5 (20)	9 (11)	0.316
Cardiovascular disease	6 (6)	1 (4)	5 (6)	1.0
Respiratory disease	5 (8)	1 (4)	4 (5)	1.0
Chronic renal failure	8 (8)	3 (12)	5 (6)	0.394
Liver cirrhosis	3 (3)	1 (4)	2 (3)	0.565
Rheumatologic disease	1 (1)	1 (4)	0	0.240
Neurodegenerative disease	6 (6)	2 (8)	4 (5)	0.628

Clinical manifestation				
ED visits on weekends	36 (35)	9 (36)	27 (34)	0.868
Mental change	6 (6)	3 (12)	3 (4)	0.148
Fever > 24 hr	33 (32)	4 (16)	27 (34)	0.343
Presence of central venous catheter	17 (16)	4 (16)	13 (16)	1.0
Hospital-acquired type	9 (9)	2 (8)	7 (9)	1.0

Solid tumor, no. (%)	69 (66)	14 (56)	55 (70)	0.211
Breast	34 (33)	5 (20)	29 (37)	0.146
Gastrointestinal tract	14 (13)	4 (16)	10 (13)	0.738
Lung	7 (7)	0	7 (9)	0.191
Hepatobiliary tract	6 (6)	2 (8)	4 (5)	0.628
Ovary	3 (3)	0	3 (4)	1.0
Others	5 (5)	3 (12)	2 (3)	0.088
Stage IV	34	10	24	0.712

Hematological malignancy, no. (%)	35 (34)	11 (44)	24 (30)	
Leukemia	11 (11)	5 (20)	6 (8)	0.211

History of Chemotherapy				
none	3	1	2	
oral chemotherapy	5	3	2	
Intravenous chemotherapy	96	21	75	
Latency > 2 months	7	3	4	
< 2months, days	12.5 (10-15)	11 (7.7-13.0)	12.5 (10-15)	0.043^∗^

^*∗*^p <0.05, ^∗∗^p<0.01 ^∗∗∗^p<0.001: significant change from baseline values.

ED: emergency department.

**Table 2:** Comparison of vital signs and laboratory findings of 25 case patients and of 79 control patients with neutropenic fever in the emergency department.

Characteristics	Total (n = 104)	Case (n = 25)	Control (n = 79)	P value
Vital signs on presentation				
SBP, mm Hg	119.1 ± 21.6	106.7 ± 24.6	122 (110-135)	0.001^∗∗^
DBP, mm Hg	70.8 ± 14.3	62.0 ± 14.1	73.6 ± 13.3	0.0003^∗∗∗^
PR, beats/min	110 (92-128)	119.7 ± 18.9	106.0 (88.5-124.7)	0.013^∗^
RR, breaths/min	18 (18-20)	20 (18-24)	18 (18-20)	0.252
Body temperature, °C	38.4 (38-38.9)	38.5 (38.0-38.1)	38.3 (38.0-38.7)	0.649
Saturation, %	97 (96-98)	96.0 (93.1-97.0)	97 (96-98)	0.001^∗∗^

qSOFA	0.49 ± 0.63	0.88 ± 0.78	0.36 ± 0.53	0.0003^∗∗∗^

Complete blood cell counts				
Leukocyte count, x10^9^cells/mL	1120(615-1570)	710 (462-1473)	1170 (702-1625)	0.056
ANC	205 (84-567)	174 (67-335)	222 (101-666)	0.087
Profound neutropenia, no. (%)	29 (28)	10 (25)	19 (24)	0.123
Hemoglobin, g/dL	9.6 ± 2.1	8.1 ± 2.2	10.3 (9.1-11)	< 0.0001^∗∗∗^
Platelet, x10^3^/*μ*l	95 (52-178)	52 (27-114)	123 (68-188)	0.002^∗∗^

Other laboratory findings				
CRP, mg/dL	5.5 (3-12.6)	10.2 (6.5-17.7)	4.0 (2.2-9.5)	< 0.0001^∗∗∗^
Lactic acid, mmol/L	1.9 (1.4-2.5)	2.15 (1.4-5.0)	1.75 (1.4-2.35)	0.126
Glucose, mg/dL	131 (112-161)	159 ± 55.4	129 (112-147)	0.106
Total bilirubin, mg/dL	0.7 (0.5-1.1)	0.9 (0.5-1.2)	0.6 (0.4-0.9)	0.029^∗^
Creatinine, mg/dl	0.77 (0.62-0.96)	0.97 (0.74-2.01)	0.72 (0.62-0.89)	0.001^∗∗^
Albumin, g/dL	3.5 (3.2-3.8)	3.1 ± 0.4	3.7 (3.3-3.8)	0.0004^∗∗∗^

Arterial blood gas				
pH	7.47 (7.44-7.49)	7.46(7.43-7.49)	7.47 ± 0.03	0.124
PCO_2_, mm Hg	31.5 ± 5.7	30.6 ± 8.1	31.9 ± 4.6	0.334
PO_2_, mm Hg	81.2 (73.7-94.8)	78.7 (69.6-95.9)	81.6 (78.8 -91.4)	0.402
HCO_3_^−^, mmol/L	22.7 (20.8-24.9)	20.2 ± 4.8	23.4 ± 2.8	0.0002^∗∗∗^
SpO_2_, mm Hg	96.7 (95.3-97.7)	96.2 (94-97.5)	96.8 (95.5-97.7)	0.150

^*∗*^p <0.05, ^∗∗^p<0.01 ^∗∗∗^p<0.001: significant change from baseline values.

qSOFA: quick sepsis-related organ failure assessment; ANC: absolute neutrophil count; CRP: C-reactive protein.

**Table 3:** Comparison of source of infection and treatment outcome of 25 case patients and of 79 control patients with neutropenic fever in the emergency department.

Characteristics	Total (n = 104)	Case (n = 25)	Control (n = 79)	P value
Source of infection, no. (%)	26 (25)	13 (52)	13 (16)	0.0004^∗∗∗^
Respiratory tract	9 (9)	3 (12)	6 (8)	0.446
Urinary tract	6 (6)	1 (4)	5 (6)	1.0
Gastrointestinal tract	2 (2)	1 (4)	1 (1)	0.424
Hepatobiliary tract	3 (3)	1 (4)	2 (3)	0.565
Skin and soft tissue	58 (26)	6 (24)	52 (66)	0.0003^∗∗∗^
Unknown				

Bacteremia, no (%)	12 (12)	6 (24)	6 (8)	0.035^∗^
Combination therapy, no (%)	21 (20)	8 (32)	13 (16)	0.093
Time from ED visit to antibiotics, min	107 (83-135)	101 (85-119)	110.5 (83-139)	0.324
Inappropriateness of antibiotics, no (%)		1 out of 7	1 out of 11	1.0
Use of G-CSF, no. (%)	58 (56)	14 (56)	44 (56)	0.978

Use of vasopressor, no. (%)	16 (15)	14 (56)	2 (3)	< 0.0001^∗∗∗^
Intubation, no. (%)	6 (6)	6 (24)	0	
ICU care, no. (%)	20 (19)	20 (80)	0	
DNR order, no. (%)		7 (28)	0	

Hospital days	7 (5-12)	11 (6.7-22)	7 (4-10.7)	0.010^∗^
In-hospital death, no (%)	12 (12)	12 (48)	0	

^*∗*^p <0.05, ^∗∗^p<0.01 ^∗∗∗^p<0.001: significant change from baseline values.

ED: emergency department; G-CSF: granulocyte-colony stimulating factor; ICU: intensive care unit; DNR: do not resuscitate.

**Table 4:** Logistic regression analysis of prognostic factors in cancer patients with febrile neutropenia in the emergency department.

Characteristics	Simple logistic analysis		Multiple logistic analysis	
	OR (95% CI)	P value	OR (95% CI)	P value
Vital signs on presentation				
SBP, mm Hg	0.95 (0.93-0.98)	0.0005^∗∗∗^		
DBP, mm Hg	0.94 (0.90-0.97)	0.0003^∗∗∗^		
PR, beats/min	1.02 (1.00-1.05)	0.011^∗^		
Saturation, %	0.84 (0.71-1.00)	0.002^∗∗^		

qSOFA			4.62 (1.17-18.22)	0.285^∗^

Laboratory findings on presentation				
Hemoglobin, g/dL			0.51 (0.33-0.78)	0.002^∗∗^
Platelet, x10^3^/*μ*l	0.99 (0.98-0.99)	0.002^∗∗^		
CRP, mg/dL	1.09 (1.03-1.15)	0.0006^∗∗∗^		
Total bilirubin, mg/dL			7.69 (1.29-45.8)	0.025^∗^
Creatinine, mg/dl	3.52 (1.48-8.37)	0.0004^∗∗∗^		
Albumin, g/dL	0.17 (0.06-0.50)	0.0004^∗∗∗^		
Bacteremia, no (%)	3.84 (1.11-13.2)	0.036^∗^		

Arterial blood gas				
HCO_3_^−^, mmol/L	0.77 (0.66-0.90)	0.0002^∗∗∗^		

Infection focus, no. (%)				
Respiratory tract			29.65 (3.81-230.7)	0.0012^∗∗^

^*∗*^p <0.05, ^∗∗^p<0.01 ^∗∗∗^p<0.001: significant change from baseline values.

CI: confidence interval; OR: odds ratio.

## Data Availability

The data used to support the findings of this study are available from the corresponding author upon request.

## Additional Points

*Key Messages.* (1) The qSOFA can be a useful bedside tool for patients with cancer who present with febrile neutropenia at the ED.
